# Cochrane systematic reviews and co-publication: dissemination of evidence on interventions for ophthalmic conditions

**DOI:** 10.1186/s13643-015-0104-5

**Published:** 2015-09-22

**Authors:** Xue Wang, Barbara S. Hawkins, Kay Dickersin

**Affiliations:** Department of Epidemiology, Johns Hopkins Bloomberg School of Public Health, The Johns Hopkins University, Baltimore, Md 21205 USA; The Wilmer Eye Institute, Johns Hopkins School of Medicine, Baltimore, Md 21287 USA

**Keywords:** Cochrane reviews, Co-publication, Citation

## Abstract

**Background:**

Systematic reviews of interventions provide a summary of the evidence available on intervention effectiveness and harm. Cochrane systematic reviews (CSRs) have been published electronically in the Cochrane Database of Systematic Reviews (CDSR) since 1994, and co-publication (publication of a Cochrane review in another journal) has been allowed since that time, as long as the co-publishing journal has agreed to the arrangement. Although standards for co-publication were established in 2008, the frequency of co-publication and adherence to the standards have remained largely unexamined. Our objective was to examine the frequency of co-publication of Cochrane Eyes and Vision Group (CEVG) reviews, adherence to the co-publication policy, the relative numbers of citations of the two modes of publishing, and differences in times cited in CSRs with and without a co-publication.

**Methods:**

We identified all CEVG reviews published by May 30, 2014 in *The Cochrane Library*. Using keywords from the title, author names, and “Cochrane Eyes and Vision Group”, we searched Google Scholar, Web of Science, Scopus, and PubMed databases to identify possible co-publications. We also emailed contact authors of all identified CEVG reviews to ask them whether they had published their CSR elsewhere. We compared each co-publication to the corresponding CEVG review for adherence to the Cochrane Policy Manual (dated June 10, 2014). We recorded the number of times each CEVG review and each co-publication had been cited by others according to Google Scholar, Web of Science, and Scopus, as of June 11, 2014.

**Results:**

We identified 117 CEVG reviews;19 had been co-published in 22 articles. Adherence to Cochrane policy on co-publication was moderate, with all authors complying with at least one of four requirements we addressed. Co-publications were cited more often than the corresponding CEVG reviews; CEVG reviews with at least one co-publication were cited approximately twice as often as CEVG reviews without a co-publication. The number of citations varied considerably depending on whether the CEVG review had a co-publication or not.

**Conclusions:**

The findings support encouraging co-publication while maintaining the primacy of the Cochrane systematic review. Support for co-publication may be tempered by other factors such as the possibility that CEVG reviews with a co-publication covered more clinically important and timely topics than those without a co-publication. Assuming that citations are a valid measure of dissemination effectiveness, the 15-year CEVG experience with co-publication of systematic reviews suggests that Cochrane authors should be encouraged to co-publish in traditional medical journals.

**Electronic supplementary material:**

The online version of this article (doi:10.1186/s13643-015-0104-5) contains supplementary material, which is available to authorized users.

## Background

Systematic reviews of interventions aim to provide a summary of the evidence available to address a research question about intervention effectiveness and harm. Since 1994, Cochrane Systematic Reviews (CSRs) have been published electronically in the Cochrane Database of Systematic Reviews (CDSR), which is included in *The Cochrane Library*, and they are updated as needed. To attract authors and maximize dissemination of the reviews, the Cochrane Collaboration encourages authors to consider the option of “co-publishing” their CSRs in traditional journals alongside their CDSR publication.

Both the Cochrane Editorial and Publishing Policy Resource (referred to as the “Policy Manual” hereafter) [1] and *The Cochrane Handbook* [2] have delineated co-publication standards. The eight requirements for co-publication are summarized in Additional file 1; only four (one of which addresses two issues) can be examined for adherence in the text of a co-publication.

Whether to pursue co-publication in a traditional journal typically is the choice of CSR authors; in some cases, co-publication may be suggested by Cochrane review group editors when they deem the topic to be timely and of special interest to clinicians and patients. For example, the Cochrane Skin Group, which has co-published about one third of their CSRs, encourages the practice [3].

The objective of our cross-sectional study was to examine the frequency of co-publication of Cochrane Eyes and Vision Group (CEVG) reviews, adherence to co-publication policy, whether co-publication of CSRs in the field of eyes and vision has been associated with additional citation of CSRs, and whether CSRs with co-publications have been cited more often than CSRs without co-publications. To our knowledge, these issues have not been addressed for CSRs published by any other Cochrane review group. 

## Methods

For Cochrane Collaboration-related projects, the Johns Hopkins Bloomberg School of Public Health allows us to query authors about their studies without specific ethics approval. Otherwise, no humans were involved in this research project, and we did not request ethics approval for any portion of the project.

We selected for study all CEVG reviews published in the CDSR as of May 30, 2014. We identified all potential co-publications using a three-step strategy. In the first step, performed in January and February 2013, for each CEVG review, we searched Web of Science and Scopus citations for title words and the first author’s surname. In the second step, we searched Google Scholar and PubMed using the term “Cochrane eyes and vision group”. In neither the first nor the second step did we limit our searches by language of publication. In the third step, performed in March 2013, we surveyed the contact authors of all CEVG reviews via email to ask whether they had published elsewhere on the topic of their CSR, specifically, whether they had co-published a Cochrane review. When contact authors appeared on more than one CSR, we asked about all reviews on which their names appeared. We performed final searches of the CDSR and citation databases on June 11, 2014. We paired the most recent versions of the CEVG reviews with co-publications, recognizing that multiple CSRs can contribute to a single co-publication and that one CSR may lead to two or more co-publications on the same or different aspects of the research question.

We used a pre-tested online data collection form, created in Google Forms, to enter the study information for each CEVG review-co-publication pair or group. The online data collection form was pre-tested by collecting data from several randomly selected co-publications and corresponding CEVG reviews to ensure the completeness of data collection. Data extracted include the following: study characteristics such as journal of the co-publication and Journal Impact Factor (JIF), comparison of publication dates between co-publication and corresponding CSR, authorship, number of included studies, and fulfillment of each Policy Manual requirement for the co-publication that we could evaluate. Details of data extraction are provided in Additional file 2.

On June 11, 2014, we searched Google Scholar, Web of Science, and Scopus for each review and each co-publication and recorded the number of times each one had been cited. Whenever multiple versions of a CEVG review were found, we checked the citation list of each co-publication to make sure that it cited no more than one version of the same CSR. To obtain the total number of times each review was cited, we counted each time a citation was made to any version of the review. When two or more co-publications had emanated from one CEVG review topic, we summed the times cited for all co-publications. We also recorded the number of times each co-publication and corresponding CEVG review had been cited by the review authors themselves (i.e., “self-citation”). We then compared the number of citations for each co-publication and its corresponding CEVG review, identified by the three citation resources, using the Wilcoxon matched-pairs signed-rank test.

We compared the numbers of citations to CEVG reviews with co-publications to the numbers of citations of CEVG reviews without co-publications, using the Wilcoxon rank-sum test, for each of the three citation resources, including and excluding self-citations. All statistical analyses were conducted using Stata 12 (StataCorp. 2011. Stata Statistical Software: Release 12. College Station, TX: StataCorp LP).

The Policy Manual requirements for co-publication of CSRs are given in Additional file 1. Specifically, the journal version must faithfully reflect the Cochrane version, indicate that it is a secondary publication, acknowledge support from the CRG, and cite the Cochrane review. We were able to evaluate adherence to four of the requirements based on comparison of CSRs and corresponding co-publications while noting that one is a two-part requirement.

## Results

### Characteristics of co-publications

As of May 30, 2014, 117 CEVG reviews had been published in the CDSR. By searching citation and bibliographic databases, we identified 21 co-publications corresponding to 18 CEVG reviews. From our survey of 88 contact authors of 105 CEVG reviews published by March 2013 to which 79 (90 %) of the contact authors responded, we identified one co-publication from one CSR not identified by our search. Therefore, we identified a total of 22 co-publications corresponding to 19 CEVG reviews. One co-publication [4] was generated from two CEVG reviews [5, 6] and four CEVG reviews [7–10], each had two co-publications [11–18]; there was one co-publication from each of the remaining 13 CEVG reviews [19–44]. Characteristics of the 22 co-publications are summarized in Table 1.

**Table 1:** Characteristics of 22 co-publications matched to 19 Cochrane Eyes and Vision Group systematic reviews

Study characteristics	*N*	(%)
Co-publication content compared to the CEVG review		
Identical^a^	3	(14 %)
Similar but not identical^a^	14	(64 %)
Abridged^a^	3	(13 %)
Other^b^	2	(9 %)
Journal of co-publication		
Ophthalmology journal	12	(55 %)
Indexed in Web of Science or Scopus as of publication date	11/12	
General or other medical journal	10	(45 %)
Indexed in Web of Science or Scopus as of publication date	7/10	
Authorship of co-publication		
Identical to CEVG review	11	(50 %)
Same authors, different order	3	(14 %)
One author added or removed	8	(36 %)
Co-publication timing		
Before the CEVG review^c^	5	(23 %)
Within 2 years after CEVG review	14	(63 %)
More than 2 years after CEVG review	3	(14 %)
Co-publication based on		
Original review	15	(68 %)
Updated review	7	(32 %)
Citation of the CEVG review by the co-publication		
Cited	12	(55 %)
Not cited, but CEVG review mentioned in the text	4	(18 %)
Not cited or mentioned	6	(27 %)
Country of affiliation of co-publication first author		
UK	16	(73 %)
USA	6	(27 %)
Number of included studies ^d^		
Same as CEVG review	16	(73 %)
Some overlap with CEVG review	6	(27 %)

^a^ *Identical* is exact copy of CSR, *Similar* would be applied, for example, when the co-publication has a shorter methods section, and *Abridged* would be applied, for example, when the co-publication is a summary of the major findings from CSR

^b^Of two co-publications classified as “Other”, one co-publication-CSR pair had no included studies and the authors discussed the characteristics of the condition; the second co-publication reported a subset of the interventions described in the CEVG review

^c^Five CEVG reviews were published after the co-publications

^d^Number of included studies in co-publication: 4 had 0 studies, 6 had 1–4 studies, 6 had 5–9 studies, and 6 had ≥10

**Fig. 1:**
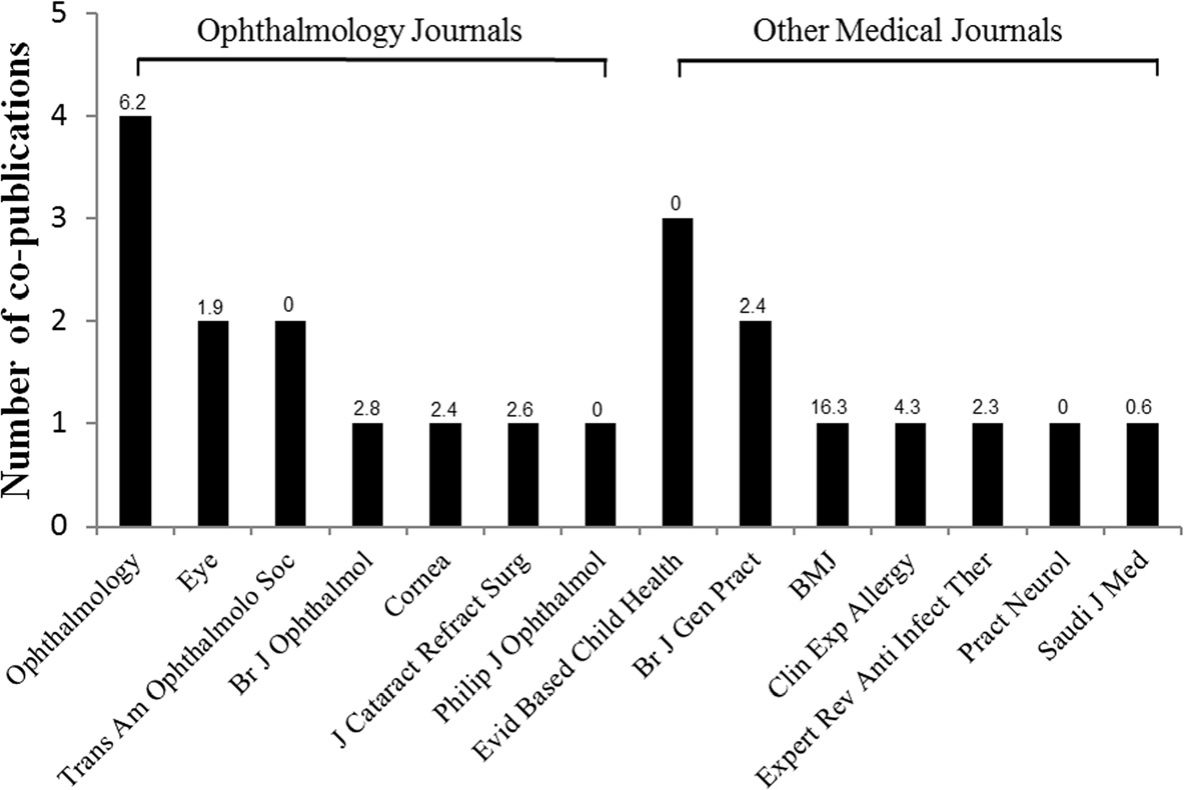
Journals of co-publication, by type of journal and Journal Impact Factor (JIF). Journal Impact Factor (JIF; rounded) as of March 9, 2015, is given at the top of each *bar*

Twelve of 22 (55 %) co-publications were in ophthalmology journals, and 10/22 (45 %) were published in general medical or other journals (Table 1, Fig. 1). Among the 12 co-publications in seven ophthalmology journals, two reviews were published in ophthalmology subspecialty journals. Most CEVG co-publications (15/22; 86 %) appeared in journals indexed in Google Scholar, Web of Science, or Scopus at the time of publication. The median JIF of the journals of co-publication was 1.83; the JIF ranged from 0 (journals without JIFs) to 17.215 (*BMJ*). Among the 12 journals, only one is open-access (*Saudi Medical Journal*).

**Fig. 2:**
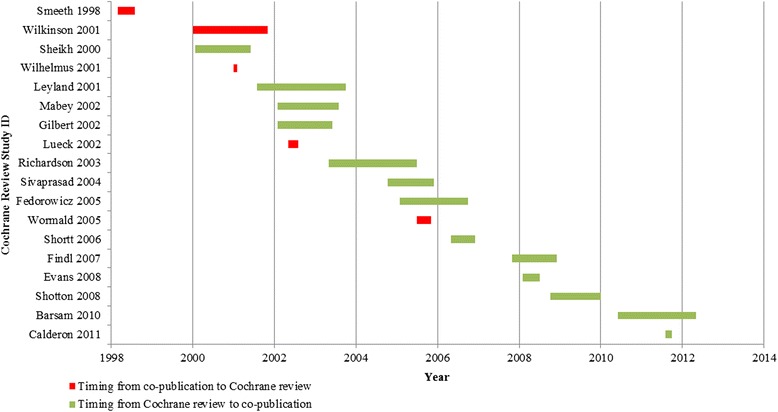
Timing of publication of 18 pairs of CEVG reviews and corresponding co-publications. Note: *green bars* indicate co-publications published after the CEVG review; *red bars* indicate co-publications published before the CEVG review

A majority of co-publications (63 %) were published within 2 years after publication of the CEVG review. However, five co-publications [17, 30, 40, 42, 44] were published before the corresponding CEVG review. The time difference between each CEVG review and co-publication is shown in Fig. 2. A majority (62 %) of the co-publications were based on the original (first) version of the CEVG review.

Among the 18 co-publications with at least one included study, 13 (72 %) cited all studies included in the CEVG review, three (17 %) cited some of the included studies [14, 26, 32], and two (11 %) did not cite any of the included studies [16, 40].

### Fulfillment of evaluable Policy Manual requirements for co-publication

*1. Reflect the data and interpretation of the CSR faithfully.* (Requirement 5, see Additional file 1)

All 22 co-publications (100 %) had the same conclusion (i.e., interpretation agreed) as the corresponding CEVG review, although 6/22 (27 %) included a different number of studies (i.e., data differed) from the CEVG review (Additional file 3 lists the reasons).

*2. Indicate that the journal version is a secondary publication (complete republication, abridged republication, complete translation, or abridged translation).* (Requirement 6)

Thirteen of the 22 co-publications (59 %) indicated that the co-publication was a secondary publication of the CEVG review (see Additional file 4).

*3. Acknowledge the support of the Cochrane Review Group in publishing the CSR.* (Requirement 7)

Half of the co-publications (11/22, 50 %) acknowledged the support of the CEVG.

*4. Cite the corresponding CSR in the co-publication reference list and, when applicable (*i.e.*, co-publication was published before the most recent version of the CSR), list the co-publication in the section “other published versions of this review” of the corresponding CSR.* (Requirement 8)

Eleven of 22 (50 %) co-publications cited the corresponding CEVG review; 6 of 19 (32 %) CEVG reviews included the co-publication information in the “other published versions of this review” section of the CSR (Table 1).

### Number of times cited

As shown in Table 2, the 22 co-publications had been cited approximately 3.5 times as often as the corresponding CEVG reviews, based on information in the Web of Science and Scopus. Based on information from Google Scholar, the number of citations to co-publications and CEVG reviews were similar. Self-citations accounted for fewer than 10 % of all citations and did not influence these findings.

**Table 2:** Numbers of citations of co-publications and corresponding CEVG reviews, as of June 11, 2014

Co-publication (*N* = 22)	No of citations, Google Scholar	No. of citations, Web of Science	No. of citations, Scopus	CEVG review (*N* = 19)	No. of citations, Google Scholar	No. of citations Web of Science	No. of citations, Scopus
Barsam 2012 [20]	3	1	1	Barsam 2010 [19]	23	7	4
Buehl 2008 [22]	40	22	22	Findl 2007 [21]	100	43	37
Calderon 2011 [11]	32	20	21	Calderon 2011 [7]	19	7	1
Calderon 2012 [12]	N/A^a^	N/A^a^	0				
Evans 2008 [4]	82	37	49	Evans 2006 [5]	111	45	14
				Evans 2008 [6]	28	33	35
Fedorowicz 2006 [24]	12	8	6	Fedorowicz 2005 [23]	47	1	0
Gnanaraj 2005 [34]	12	3	9	Richardson 2003 [33]	32	4	12
Leyland 2003 [28]	183	109	130	Leyland 2001 [27]	87	26	18
Lueck 2002 [30]	11	N/A^a^	8	Lueck 2002 [29]	77	0	10
Mabey 2003 [32]	N/A^a^	N/A^a^	5	Mabey 2002 [31]	69	6	9
Sheikh 2001 [13]	81	43	52	Sheikh 2000 [8]	158	33	21
Sheikh 2005 [14]	54	25	30				
Shortt 2006 [36]	69	39	50	Shortt 2006 [35]	7	0	3
Shotton 2009 [15]	N/A^a^	N/A^a^	N/A^a^	Shotton 2008 [9]	4	7	8
Shotton 2009 [16]	N/A^a^	N/A^a^	N/A^a^				
Sivaprasad 2005 [38]	43	22	31	Sivaprasad 2005 [37]	83	2	8
Smeeth 1998 [40]	40	28	34	Smeeth 1998 [39]	44	7	4
Stanford 2003 [26]	79	36	48	Gilbert 2002 [25]	31	0	0
Wilhelmus 2000 [42]	42	N/A^a^	28	Wilhelmus 2001 [41]	23	7	4
Wilkinson 1999 [17]	10	N/A^a^	6	Wilkinson 2001 [10]	36	0	2
Wilkinson 2000 [18]	90	38	50				
Wormald 2005 [44]	N/A^a^	N/A^a^	N/A^a^	Wormald 2005 [43]	146	35	34
Median	42	26.5	28		40	7	8
Range	3 to 183	1 to 109	0 to 130		4 to 158	0 to 78	0 to 49
Wilcoxon matched-pairs signed-rank test^b^	*P* = 0.32	*P* = 0.07	*P* = 0.004				

All numbers cited include self-citations

^a^N/A indicates that the article was not found in Google Scholar, Web of Science or Scopus, sometimes because the journal was not indexed

^b^The Wilcoxon matched-pairs signed-rank test was used to test the difference between the number of citations to each co-publication and its corresponding CEVG review in each database (Google Scholar, Web of Science, and Scopus, respectively). We considered a value of *P* < 0.05 to indicate a statistically significant difference in numbers of citations between a co-publication and its corresponding CEVG review

The 19 CEVG reviews with at least one co-publication had been cited twice as often as the 98 CEVG reviews without a co-publication, or more often when we considered the data retrieved from Google Scholar (Table 3). Numbers of citations documented by Web of Science and Google Scholar differed between co-published CEVG reviews and CEVG reviews without co-publications but were similar within Scopus (Table 3).

**Table 3:** Numbers of citations to CEVG reviews without versus with one or more co-publications

Citations from three sources	CEVG reviews without a co-publication	CEVG reviews with ≥1 co-publication	*P* value from Wilcoxon rank-sum test
Number of reviews	98	19	
Times cited in Google Scholar, median (IQR)	18 (7 to 36)	44 (23 to 87)	*P* = 0.0003
Times cited in Web of Science, median (IQR)	3 (0 to 7)	7 (1 to 33)	*P* = 0.04
Times cited in Scopus, median (IQR)	5 (1 to 11)	9 (2 to 18)	*P* = 0.20

The numbers of citations are those found on June 11, 2014

*CEVG* Cochrane Eyes and Vision Group, *IQR* interquartile range

## Discussion

Only a small proportion of CEVG reviews (19/117, 16.2 %) had been co-published based on information available as of May 2014. The percentage of CSRs co-published has changed little since 2007 (10/51; 19.6 %) [45]. We do not know the reason for the relatively low percentage; despite co-publication agreements with many journals, many authors (and even editors) may not be aware of them. Following a survey of authors and editors in the field of dermatology, Cochrane authors recommended that authors of CSRs who wish to co-publish their reviews ‘minimize frustration’ by first considering journals that have co-published other Cochrane reviews [3].

Physicians and researchers who do not use or do not have easy access to the CDSR may not be aware of important and up-to-date evidence synthesis it contains, and co-publication could provide this access. Does co-publication represent a form of duplicate publication? We do not think so, as the Cochrane review is unlikely to be “the same as” a co-publication, in that it is likely to be much longer and its structure follows a specified Cochrane format. Thus, one view could be that Cochrane is helping to reduce research waste by its co-publication policy, by making it more likely that the evidence gets to clinicians and others who can use it. It would be worth discussing whether Cochrane could do even more to reduce research waste by suggesting co-publication in open-access journals.

For the same review topic, the co-publication had been cited more often than the CEVG review, although the difference was statistically significant only within the Scopus database (Table 2). Although Web of Science and Scopus produced lower citation estimates than Google Scholar, nevertheless CEVG reviews with co-publications also had been cited about twice as often as those reviews without co-publications (Table 3).

The Policy Manual requirements for co-publication typically had not been fulfilled by the authors of CEVG reviews. All co-publications drew the same conclusions about treatment effectiveness as the corresponding CEVG reviews, including six that had different numbers of included studies. However, when co-publications appeared after or at the same time as the CEVG review (19/22 or 86 % of co-publications), only half of the co-publications acknowledged the support of the CEVG or cited the CEVG review. We recommend that the CEVG and other Cochrane Review Groups who wish to encourage co-publication monitor co-publications for fulfillment of the Cochrane policy. Adherence to co-publication policies of both Cochrane and individual journals should be important to editors of journals with co-publication agreements with Cochrane [46].

Thus, co-publication, when the CSR is cited properly, may bring attention to Cochrane reviews and increase dissemination. Our findings support encouraging co-publication while maintaining the primacy of the Cochrane systematic review. Support for co-publication may be tempered by other factors, of course, such as the reasons for co-publication, a topic we have not explored in this study. For example, it is possible that CEVG review authors are more likely to pursue a co-publication for more clinically important and timely topics or for more complex systematic reviews and meta-analyses. In addition, we do not know whether any authors of CEVG reviews prepared their findings for co-publication only to have their manuscript rejected by editors who saw no need to use journal space to publish information already publicly available.

As far as we know, the notion of co-publication, *per se*, is unique to Cochrane systematic reviews. This is likely because the Cochrane Collaboration has, from its beginning in 1993, wanted to attract authors who sought publishing outlets in addition to *The Cochrane Library*. The Agency for Healthcare Research and Quality, another commissioner of systematic reviews, publishes the Effective Healthcare Programs full reviews on its website (http://www.ahrq.gov/research/findings/evidence-based-reports/) and encourages authors to publish a shorter version elsewhere as well (e.g., http://www.jclinepi.com/content/jce-AHRQ-Series).

Discussion within Cochrane as to whether authors should be encouraged to co-publish in other journals has mainly concerned broader dissemination and providing opportunities for attracting authors. For example, it is possible that co-publications will be read and cited by physicians and scientists and thus may lead readers to consult the full Cochrane review; if this assumption is correct, co-publication is one way to disseminate findings of Cochrane reviews and to increase their influence. On the other hand, co-publications may attract attention to the co-publishing journal and may not draw attention to Cochrane reviews; if this assumption is correct, Cochrane reviews may be less cited and less influential than the co-publications. Furthermore, if co-publications are cited more often than Cochrane reviews, review authors may be motivated to publish first in journals other than *The Cochrane Library*. Some review groups have asked whether it is a good idea to write to authors of systematic reviews published elsewhere to convert their reviews to CSRs. We found little evidence that this strategy had been employed in the CEVG, since in only 3/22 cases of co-publication of CEVG reviews was there evidence that the CSR was completed after the co-publication [10, 39, 41].

We observed a tendency to increased citation of co-publications over the CEVG reviews, regardless of the database used to identify citations; however, the numbers of citations identified by each source varied. Web of Science had the smallest number of citations among the three databases, and Google Scholar had the greatest number of citations for both CEVG reviews and corresponding co-publications. We noted that Google Scholar took into account the numbers cited for multiple versions of CSRs and had not removed duplicate citations. We expect that there are other differences between databases that account for discrepancies in numbers of citations of both co-publications and CSRs, but we did not seek to understand these discrepancies in any depth.

Although we imposed no language restrictions on our search, we did not attempt to search any non-English database. Given this fact, the lack of response from ten contact authors to our inquiry and domination of Web of Science, Scopus, Google Scholar, and PubMed by English-language journals, it is possible that we missed co-publications not in the English language. We also did not survey review authors regarding their reasons for co-publishing their CEVG review or choosing not to do so.

The JIF, a ratio reflecting the number of citations to articles published in a specific journal during the previous 2 years divided by the total number of citable articles published in the journal, is considered by many publishers, readers, authors, and reviewers to be a marker of a journal’s importance. The CDSR received its first impact factor in 2007 (4.654) and its most recent JIF in 2014 (6.032). This may not reflect the “impact” of CEVG reviews however. To understand how the JIF and other considerations have affected authors’ decisions to co-publish or not, further surveys of review authors, both those who have co-published and those who have not, would be required. Editors also may be a factor in co-publication. A 2008 survey of dermatology journal editors found that most (6/11) editors who had co-published Cochrane reviews believed that co-publication of the review could increase their journal’s impact factor [3].

The Cochrane Back Review Group also recently examined co-publication and had similar findings to our own [47]. With 62 published reviews, as of Issue 4, 2013 of *The Cochrane Library*, and 59 co-publications (some Cochrane reviews had more than one co-publication), a greater proportion of their reviews had been co-published compared to the CEVG reviews. For the ten most frequently cited reviews from the Cochrane Back Review Group, the number of citations to co-publications were generally the same or greater than to the review itself; overall, the co-publications had been cited about two times more often than the ten top-cited Cochrane reviews.

## Conclusions

Only about one in seven CEVG reviews have been co-published, but, when they have been, co-publications have been cited more often than the corresponding CEVG reviews. If one accepts citations to be a valid measure of dissemination effectiveness, the CEVG experience with co-publication of systematic reviews during the past 15 years suggests that authors of CEVG reviews who wish to increase dissemination of their findings should co-publish them in traditional medical journals. Cochrane review groups may be wise to invest some resources in co-publication.

## Additional files

Additional file 1:
**Requirements for co-publication given in the Cochrane Policy, as of June 10, 2014.**
Additional file 2:
**Data extraction form.**
Additional file 3:
**Reasons six co-publications had a different number of included studies compared to the CEVG review.**
Additional file 4:
**Statements of secondary publication in 13 CEVG co-publications.**

## Abbreviation

CDSR: Cochrane Database of Systematic Reviews
CEU: Cochrane Editorial Unit
CEVG: Cochrane Eyes and Vision Group
CRG: Cochrane Review Group
CSR: Cochrane Systematic Reviews
IQR: interquartile range
JIF: Journal Impact Factor
WHO: World Health Organization
