# Supplementary material for: Cochrane systematic reviews and co-publication: dissemination of evidence on interventions for ophthalmic conditions
Source: Syst Rev. 2015 Sep 22;4:118. doi: 10.1186/s13643-015-0104-5 (PMC4580360; doi:10.1186/s13643-015-0104-5)
Supplement: Additional file 1: — Requirements for co-publication given in the Cochrane Policy, as of June 10, 2014. [file 13643_2015_104_MOESM1_ESM.pdf]

Additional file 1 -- Requirements for co-publication given in the Cochrane Policy, as of 10 June 2014

1. For all instances of co-publication, the authors should approach the CRG's Managing Editor using the 'Permission to co-publish form'. Permission will not be unreasonably withheld.
2. If the authors propose publishing an abridged version in a journal:
   a. before publication in the CDSR (prior publication) – the CRG will refer the case to the CEU for permission.
   b. at the same time as publication in the *CDSR* (simultaneous publication) – the CRG will refer the case to the CEU for permission or inform the CRG if there is an existing co-publication agreement with the journal.
   c. after publication in the *CDSR* (post-publication) but either the journal has not yet signed a Cochrane co-publication agreement or if the journal appears on the list of high-profile journals – the CRG will refer the case to the CEU for permission. The CEU will consult with the CRG, the authors, and the journal, as necessary. The CEU will ask Wiley to ensure that the journal signs a Cochrane co-publication agreement and that permission is granted to the author. The CEU will inform the CRG of the decision, and the CRG will communicate the decision to the author.
   d. after publication in the *CDSR* (post-publication) AND the journal has already signed a Cochrane co-publication agreement– the CRG can grant permission to the author and does not need to refer to the CEU. The CRG will ensure that the CEU is aware of each permission granted by sending a copy of the completed 'Permission to co-publish' form to the CEU.
3. All co-publishing journals will be asked to sign a Cochrane co-publication agreement with Wiley. This can be a one-off agreement or an agreement to co-publish over a period of time (i.e. it does not need to be signed by the journal for each abridged review). All journals that have already signed the agreement to co-publish over a period of time are listed below. If the journal has not yet signed an agreement the CEU will instruct Wiley to ensure that an agreement is signed (in advance of submission to the journal as far as possible).
4. When submitting an abridged version of a Cochrane Review to a journal (or enquiring about submission in the case of prior or simultaneous publication), the author must make a complete statement to the journal editor to say that the article is based on a Cochrane Review (published or in progress).
5*. The journal version must faithfully reflect the data and interpretations of the Cochrane version. Where journal editing and peer review have resulted in differences in the data or interpretation, these should be discussed with the CRG, and, where appropriate, incorporated into a revised version of the Cochrane Review at the next available opportunity, and with appropriate acknowledgement to the journal's peer reviewers/editors.
6*. The title of the journal version should indicate that it is a secondary publication (complete republication, abridged republication, complete translation, or abridged translation) of the Cochrane Review, preferably by including 'Cochrane Review' or 'Cochrane Systematic Review' in the title.
7*. The support of the CRG in publishing the Cochrane Review should be acknowledged in an appropriate place in the journal version (e.g. in the Acknowledgements section).
8*. The Cochrane Review must be cited in the reference list of the journal version.
   a. A footnote should be placed on the title page of the journal version to inform readers and documenting agencies that the paper has been published previously, either in whole or in part, citing the primary publication (*CDSR*); for example: "This article is based on a Cochrane Review published in the *Cochrane Database of Systematic Reviews* (CDSR) YYYY, Issue X, DOI: 10.1002/14651858.CD00xxxx (see www.thecochranelibrary.com for information). Cochrane Reviews are regularly updated as new evidence emerges and in response to feedback, and the *CDSR* should be consulted for the most recent version of the review."
   b. The Cochrane Review published in the *CDSR* should reference the journal version in the section 'Other published versions of this review' as soon as possible after publication of the journal version or when in press.

CDSR: Cochrane Databases of Systematic Reviews; **CEU:** Cochrane Editorial Unit; **CRG:** Cochrane Review Group; **CSR:** Cochrane Systematic Review.

*Items examined in this study
