# Supplementary material for: Cochrane systematic reviews and co-publication: dissemination of evidence on interventions for ophthalmic conditions
Source: Syst Rev. 2015 Sep 22;4:118. doi: 10.1186/s13643-015-0104-5 (PMC4580360; doi:10.1186/s13643-015-0104-5)
Supplement: Additional file 2: — Data extraction form. [file 13643_2015_104_MOESM2_ESM.pdf]

Additional file 2 -- Data Extraction Form

Data item	Sub-item	Coding options
1. Journal of co-publication		
2. Journal impact factor of co-publishing journal	--	--
3. Characteristics of co-publication	3.1General content	Identical to the CSR
		Similar but not identical to CSR
		Abridged version of CSR
	3.2 Authorship	Identical to the CSR
		Similar but not identical to CSR
		Abridged version of CSR
	3.3 Publication time	Year and month
	3.4 Date of search	Year, month, and day
	3.5 Country of affiliation of first author	
	3.6 Number of included studies	
	3.7 Did the co-publication cite included studies?	
	3.7 Conclusions	
	3.8 Times cited by Web of Science (times cited by an author: “self-cites”)	
	3.9 Times cited by Scopus (self-cites)	
	3.10 Times cited by Google Scholar (self-cites)	
4.Characteristics of corresponding CSR	4.1 Publication date and issue	Year and issue
	4.2 Date of search	Year, month, day
	4.3 Version of CSR	Original review
		Updated review
	4.4 Number of included studies	
	4.5 Conclusions agree	Yes/No
5. Fulfillment of the Policy Manual requirements for co-publication	5.1 Reflect the data and interpretation of the CSR faithfully (Y/N)	Yes/No
	5.2 Indicate that the journal version is a secondary publication (complete republication, abridged republication, complete translation, or abridged	Yes/No

	translation) (Y/N)	
	5.3 Acknowledge support of the Cochrane Review Group in publishing the CSR (Y/N)	Yes/No
	5.4 Cite the corresponding CSR in its reference list if applicable (i.e., co-publication was published before the most recent version of the CSR)	Yes/No
	5.5 List the co-publication in the corresponding CSR's section "other published versions of this review" (if co-publication was published before the most recent version of the CSR)	Yes/No

CSR: Cochrane Systematic Review
