# Supplementary material for: Cochrane systematic reviews and co-publication: dissemination of evidence on interventions for ophthalmic conditions
Source: Syst Rev. 2015 Sep 22;4:118. doi: 10.1186/s13643-015-0104-5 (PMC4580360; doi:10.1186/s13643-015-0104-5)
Supplement: Additional file 3: — Reasons six co-publications had a different number of included studies compared to the CEVG review. [file 13643_2015_104_MOESM3_ESM.pdf]

Additional file 3 -- Reasons 6 co-publications had a different number of included studies compared to the CEVG review

1. The co-publication had more included studies than the CEVG review (3 co-publications):

- The co-publication was an update of the earlier co-publication of a CEVG review.
- The co-publication was published two years after the corresponding CSR, and an updated search identified additional studies.
- The co-publication included more interventions than the CSR.

2. The CEVG review had more included studies than the co-publication (3 co-publications):

- The co-publication was published before the CSR.
- The co-publication included fewer interventions or outcomes than the CSR.
- The CSR included one or more studies with unpublished data and the co-publication did not include those data.

CEVG: Cochrane Eyes and Vision Group; **CSR:** Cochrane Systematic Review
