# Supplementary material for: Cochrane systematic reviews and co-publication: dissemination of evidence on interventions for ophthalmic conditions
Source: Syst Rev. 2015 Sep 22;4:118. doi: 10.1186/s13643-015-0104-5 (PMC4580360; doi:10.1186/s13643-015-0104-5)
Supplement: Additional file 4: — Statements of secondary publication in 13 CEVG co-publications. [file 13643_2015_104_MOESM4_ESM.pdf]

**Additional file 4 -- Statements of secondary publication in 13 CEVG co-publications**

1. “This paper provides the essence of a Cochrane Review published in the Cochrane Electronic Library in January 2005."[24]
2. "An earlier version of this review has been published in The Cochrane Library."[26]
3. "This article summarises the results of two comprehensive regularly updated systematic reviews published on the Cochrane Library."[4]
4. "We have recently completed a review for the Cochrane Library on interventions for idiopathic intracranial hypertension (IIH) but could not find any relevant randomised controlled trials."[30]
5. "The review was undertaken in collaboration with the Cochrane Eyes and Vision Group…Published on the Cochrane Library of systematic reviews, April 2003."[34]
6. "This is a complete re-publication of the CDSR in the Cochrane Journal."[15]
7. "There is an extensive body of published data on both monofocal and multifocal IOLs describing largely successful outcomes. To draw some conclusions regarding the relative merits of the different IOL types, a systematic review of the best quality data (that from randomized controlled trials) was undertaken as part of the Eyes and Vision Group of the Cochrane Collaboration."[28]
8. “This paper is based on a Cochrane review most recently substantively amended in The Cochrane Library 2005, Issue 4 (see www.thecochranelibrary.com for information). Cochrane reviews are regularly updated as new evidence emerges and in response to comments and criticisms. The Cochrane Library should be consulted for the most recent version of the review.”[43]
9. "This article is based on a Cochrane Review published in the Cochrane Database of Systemic Reviews (CDSR) 2011, issue 7, Art.No: CD007685. DOI:10.1002/14651858.CD007685.pub2 (see www.thecochranelibrary.com for information). Cochrane Reviews are regularly updated as new evidence emerges and in response to feedback, and the CDSR should be consulted for the most recent version of the review."[11]
10. "This is a reprint of a Cochrane review, prepared and maintained by The Cochrane Collaboration and published in The Cochrane Library 2011, Issue 7"[12]
11. "This is a summary, including tables of key findings and quality of included trials, of a Cochrane review, published in this issue of EBCH, first published as: Shotton K, Powell C, Voros G, Hatt SR. Interventions for unilateral refractive amblyopia. Cochrane Database of Systematic Reviews 2008, Issue 4, Art. No.: CD005137. DOI: 10.1002/14651858.CD005137.pub2."[16]
12. "The systematic review presented here uses data included in the Cochrane review but considers only azithromycin and the antibiotics recommended for trachoma by the WHO."[32]
13. The title indicated the co-publication is a "Cochrane systematic review and meta-analysis update".[14]

**CEVG:** Cochrane Eyes and Vision Group; **CDSR:** Cochrane Databases of Systematic Reviews; **WHO:** World Health Organization
